# Polysaccharide and Protein Films with Antimicrobial/Antioxidant Activity in the Food Industry: A Review

**DOI:** 10.3390/polym12061289

**Published:** 2020-06-04

**Authors:** Ewelina Jamróz, Pavel Kopel

**Affiliations:** 1 Department of Chemistry, Faculty of Food Technology, University of Agriculture, ul. Balicka 122, PL-30-149 Kraków, Poland; ewelina.jamroz@urk.edu.pl; 2 Department of Inorganic Chemistry, Faculty of Science, Palacky University, 17. Listopadu 12, CZ-771 46 Olomouc, Czech Republic

**Keywords:** biopolymer films, polysaccharide, protein, active packaging materials, antimicrobial and antioxidant activity, composite materials, plant extracts, essential oil, nanofillers, food packaging systems

## Abstract

From an economic point of view, the spoilage of food products during processing and distribution has a negative impact on the food industry. Lipid oxidation and deterioration caused by the growth of microorganisms are the main problems during storage of food products. In order to reduce losses and extend the shelf-life of food products, the food industry has designed active packaging as an alternative to the traditional type. In the review, the benefits of active packaging materials containing biopolymers (polysaccharides and/or proteins) and active compounds (plant extracts, essential oils, nanofillers, etc.) are highlighted. The antioxidant and antimicrobial activity of this type of film has also been highlighted. In addition, the impact of active packaging on the quality and durability of food products during storage has been described.

## 1. Introduction

Oxidative reactions and microbiological changes are the main processes causing unwanted changes to the quality of food products, thus affecting their safety. The current challenge is to design packaging materials that would extend the shelf life of foods. Active packaging with antimicrobial and/or antioxidant activity may control these processes, while extending the shelf life of a product. This type of packaging is defined as a packaging system containing ingredients that release or absorb substances into or out of packaged food or the surrounding food environment, to extend its shelf life or to improve the condition of the packaged food. Two aspects support the use of active biopolymer films, compared to the direct incorporation of antioxidant and/or antimicrobial components: (1) the diffusion of active compounds onto the food surface can be controlled, and (2) the amount of conservatives added to food can be reduced [1]. The use of packaging materials based on biopolymers obtained from natural sources, such as proteins and polysaccharides instead of synthetic materials, contributes to reducing the accumulation of non-degradable materials. Biopolymers can be obtained from many natural sources, such as fishery or cattle farming agricultural waste [2]. These types of materials are gaining more and more attention because they are renewable, biodegradable and inexpensive. Biopolymers from natural sources consist of macromolecules, including proteins and polysaccharides, which play an important role in improving the quality of food products. Polysaccharides (chitosan, starch, agar, cellulose, furcellaran, carrageenan, etc.) and proteins (gelatin, alginate, collagen, gluten, whey protein, etc.) are starting to become an interesting alternative to synthetic packaging. However, they have many disadvantages, associated with low strength parameters and a weak barrier against water vapour and oxygen. Nonetheless, these disadvantages may be minimised by combining different biopolymers with one another, including components such as plant extracts or the addition of nanofillers to the film structure, which complement defects in the matrix [3,4].

**Figure 1:**
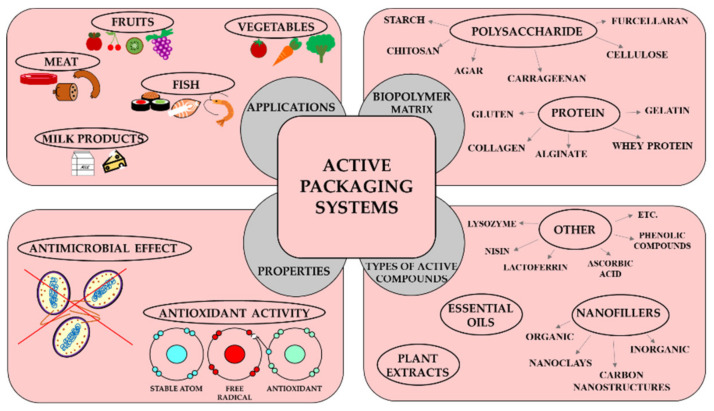
The active properties of biopolymer films as the main compounds in active packaging materials.

In this review, the current status of active packaging materials is presented on the basis of biopolymers (proteins and polysaccharides) and active ingredients (plant extracts, essential oils, nanofillers and other compounds). In the article, the antioxidant and antimicrobial properties of active packaging materials are summarised. Finally, the latest research results related to the practical use of active packaging materials in the food industry are reviewed (Figure 1).

## 2. Types of Active Compounds in Biopolymer Films and Functional Properties of Active Films

### 2.1. Antioxidant Agents

Lipid oxidation is one of the main causes of food spoilage. This is especially true in the case of products with high lipid content, such as nuts, fish and vegetable oils. Lipid oxidation results in the formation of toxic aldehydes and the loss of nutritional quality due to the degradation of polyunsaturated fatty acids [5]. Active packaging may assume antioxidant properties thanks to the presence of antioxidants, which prevent the oxidation of food. Antioxidants can further prevent lipid peroxidation via the following mechanisms:

- Preventing chain inhibition by scavenging initiating radicals
- Peroxide decomposition, so they cannot be reconverted into initiating radicals
- Breaking the chain reaction
- Reduction of localised oxygen concentrations and catalysts initiating chain bonds, such as metal ions [6,7].

The growing interest in the use of natural food additives is associated with the need to limit synthetic additives. Many synthetic antioxidants, such as organophosphate and thioester compounds, have been implemented, but due to their migration to food products and their potential toxicity, the use of such additives becomes questionable [5]. Natural antioxidants include active plant components called phenolic compounds, as well as their secondary metabolites [8]. Antioxidant packaging can work in two ways: by releasing antioxidants into a food, and by scavenging undesirable compounds that may appear during a given stage of the food oxidation process. Packaging materials which release antioxidants can discharge active substances into food in a controlled manner. In contrast, scavengers are substances that may react, modify or capture undesirable substances in the packaging environment, but these substances are not released into food [9]. In this section, the influence of adding active ingredients (plant extracts, essential oils, nanofillers and other compounds) on the antioxidant properties of the film is characterised.

#### 2.1.1. Plant Extracts

Plant extracts may be successfully used as antioxidant components in packaging materials. By means of appropriate physical and chemical processing methods, they can be extracted from various types of plant stems, roots, leaves and fruits [10]. Antioxidant activity is determined by compounds such as polyphenols, flavonoids, alkaloids and terpene substances [11,12]. Pomegranate peel contains punicalagin, which is an active component contributing to the antioxidant capacity of this fruit [13]. This type of ingredient improves the antioxidant properties of fish gelatin films [14]. The main phenolic compounds responsible for the antioxidant properties of mango skins are, among others, mangiferin, quercetin, ellagic acid and gallic acid [15]. The addition of mango peel extract to fish gelatin films caused improvement in antioxidant activity. The presence of mangiferin in mango peel extract contributes to the inhibition of auto-oxidation. Additionally, the synergistic effect of bioactive compounds can significantly influence the antioxidant nature of gelatin films [8]. The phenolic compounds—for example, benzoic acid, p-toluic acid, p-salycyclic acid, ferulic acid and aloe emodin—which are present in aloe vera gel caused an increase of the antioxidant activity of gelatin films. Moreover, the presence of lipids, proteins, enzymes and DNA in aloe vera gel affects the strong scavenging effect [16]. Peanut shell and skin extracts were added to starch–chitosan films. The antioxidant capacity of peanut extracts was greater than that of peanut shells, which may be associated with the presence of polyphenols and flavonoids, in particular, linolenic acid, rutin and 4-O-caffeoulquinic acid. The inclusion of these compounds caused a high DPPH^·^ (2,2-diphenyl-1-picryl-hydrazyl) and ABTS^+^ (2,2’-azino-bis(3-ethylbenzothiazoline-6-sulfonic acid)) radical scavenging rate [11]. Chinese hawthorn fruit extract was added to chitosan–gelatin films. The obtained films showed strong free radical scavenging ability (33.42–84.40%, measured using the DPPH^·^ method), which is associated with the presence of polyphenols (epicatechin, chlorogenic acid and procyanidin B2) [17]. Lignocellulose agricultural products, such as rice or coffee skins, are rich in polyphenols, combined with hemicellulose or lignin fractions, which have antimicrobial or antioxidant activity. Collazo-Bigliardi et al. (2019) added cellulose fibres and active fractions extracted from rice and coffee husks to thermoplastic starch films. The extracts demonstrated antioxidant activity (5.37–5.29 mg extract solids/mg DPPH^·^). Starch films with the addition of coffee husk showed greater antioxidant activity than films with rice husks [18]. Sunflower husk extract was also added to the starch films and its addition caused an increase in antioxidant activity. What is noteworthy is that the process of obtaining the film did not reduce the antiradical activity of the extract. Phenolic compounds, in particular chlorogenic acid and small amounts of coumaric and ferulic acid derivates, mono-caffeoylquinic and dicaffeoylquinic acid derivates, are responsible for the antioxidant activity of the sunflower husk extract [19]. Jirdi et al. (2019) developed gelatin films with phenolic orange peel extracts and examined the effect of drying on the quality of orange (*Citrus sinensis*) peel extracts. In research, it has been shown that fresh extracts have higher antioxidant potential [1.38-Ferric (Fe^3+^) reducing power; 95.76%—β-carotene bleaching inhibition and 99.7%—DPPH^·^ method] than thermally dried orange peel extracts. High antioxidant activity is associated with the presence of quinic acid, rutin, trans-ferulic acid, naringenin and 4,5-di-O-caffeoylquinate. Fresh extracts have antioxidant potential via acting as a hydrogen/electron donating agent, or by chelating metals, which interrupts the oxidative chain reaction [20].

#### 2.1.2. Essential Oils

Another aspect involves the incorporation of essential oils (EO) into the biopolymer matrix which can improve the antioxidant properties of packaging materials. The Food Drug Administration (FDA) considers EOs to be “generally recognized as safe” (GRAS) chemicals, which may be used as food additives in preservatives and flavouring. However, due to their intense aroma and potential side effects associated with EO sensitisation, they cannot be added directly to food. Therefore, the incorporation of essential oils into biopolymer films seems to be an interesting way to overcome these limitations [21]. There are many works in which the effects of plant essential oil addition on the antioxidant properties of biopolymer films have been investigated. Bonilla et al. (2018) incorporated ginger essential oil and eugenol into different film formulations (gelatin, chitosan, gelatin–chitosan). The gelatin–chitosan films with eugenol exhibited the best antioxidant activity (TEAC method). Eugenol has the ability to trap chain-carrying peroxy radicals (ROO^·^) by donation of the phenolic hydrogen atom reaction. The unsaturated double bond is responsible for the radical scavenging action of the eugenol radical [22]. In a different study, ginger essential oil was incorporated into gelatin and gelatin–montmorillonite films. The authors showed that the antioxidant potential of the films depended on the presence of the ginger essential oil [23]. Further, the biopolymer matrix plays the appropriate role of a carrier for active substances and, when properly designed, it allows for the most advantageous controlled release of the active compound. Xu et al. (2019) derived chitosan (CS) –gum arabic (GA) films with different biopolymer ratios (CS:GA—1:0; 1:0.25; 1:0.5; 1:1; 1:2; 1:4). Then, they added essential oil (8%) and assessed the effect of different biopolymer contents on the antioxidant activity of the film. The authors observed that the appropriate ratio of chitosan to gum arabic (from 1:0.25 to 1:2) results in higher retention and controlled release of the oil, which leads to the improved effect of oil as an antioxidant. In contrast, the design of the matrix in a ratio of 1:4 causes a decrease in antioxidant activity, which is associated with large losses of oil [24]. Similar conclusions were reached by Valizadeh et al. (2019), who added cinnamon essential oil (CEO), oleic acid (OA) and glutaraldehyde (GL) to chitosan–carboxymethyl cellulose (CS–CMC) composite films. Controlled release of the oil occurred with an antioxidant effect by trapping the essential oil droplets in the cross-linked matrix. Furthermore, it can be stated that cross-linking prevents the initial release of bioactive additives [25]. Echeverría et al. (2016) concluded that the addition of montmorillonite to soy protein films induces the polyphenols contained in clove essential oil to become more reactive against radical scavenging. In addition, the presence of nanoclay promotes the release of the essential oil’s active substances [26]. In Table 1, recent studies on biopolymer films with different antioxidant compounds are presented.

#### 2.1.3. Nanofillers

The addition of nanostructures also has a positive effect on improving the antioxidant activity of biopolymer films. Roy et al. (2020) enriched chitosan films with the addition of melanin nanoparticles. Both synthetic and natural melanin have antioxidant potential. The incorporation of melanin into chitosan films caused a significant increase in antioxidant activity (by ~163% and by ~158%, measured via the DPPH^·^ and ABTS^+^ methods, respectively). The antioxidant potential of melanin is associated with its ability to provide and receive an electron that can interact with free radicals through the process of electron transfer and, consequently, neutralise them [38]. Zhang and Zhao (2017) added rutin nanoparticles to zein films. Along with the increase in the concentration of rutin nanoparticles, the antioxidant properties of zein films also rose by ~320% (DPPH^·^ method), by ~534% (ABTS^+^ method) and by ~86% (phosphomolybdenum method) [39]. In addition, some metal nanoparticles exhibit antioxidant properties. Boughriba et al. (2020) incorporated TiO_2_–Ag nanoparticles into *Rhinobatos cemiculus* gelatin films, and noted that the addition of nanostructures increased the antioxidant activity of the film evaluated via DPPH^·^ radical scavenging activity (by ~167%) and ferrous chelating activity (by ~174%) [40].

#### 2.1.4. Other Antioxidant Compounds

There are many antioxidant additives that can be incorporated into the biopolymer matrix to provide active properties to packaging materials. Kchaou et al. (2020) added cuttlefish (*Sepia officinalis*) skin protein isolate (CSPI) and hydrolysates (CSPH) to gelatin films. Free-form CSPI showed a higher radical scavenging activity than CSPH, which may be related to the difference in their molecular weights and solubility in the ethanol solution. The authors noted that CSPH demonstrated higher antioxidant activity (measured by the DPPH method) in the gelatin matrix than in the free form, which may be attributed to the presence of protein–protein interactions or hydrogen bonds between the gelatin matrix and the added peptides [41]. Tomadoni et al. (2019) incorporated vanillin into chitosan films, which caused a significant increase in antioxidant activity [42]. Moreover, Lu and Liu (2020) added a hexahydro-β-acid/2-O-methyl-β-cyclodextrin complex to chitosan films, and the noted increase in the scavenging effect was attributed to the good antioxidant activity of hexahydro-β-acid [43]. The addition of α-tocopherol in the form of a nanocapsule suspension improved the antioxidant activity of methylcellulose films [44]. Natamycin and/or nanoemulsioned α-tocopherol were added to the whey protein films. Only films with α-tocopherol exhibited antioxidant activity (measured by the ABTS and DPPH methods), which can be attributed to the properties of α-tocopherol. This antioxidant agent can donate a hydrogen atom to quench free radicals by creating α-tocopheroxyl radicals until decolourisation of ABTS and DPPH radicals. Films with natamycin do not have antioxidant properties, while films with natamycin and α-tocopherol have a lower activity than films with α-tocopherol. Such results may be attributed to the antagonistic effects of these two agents [45]. Analogous results were observed in the study of cassava starch–chitosan films with pitanga (*Eugenia uniflora* L.) leaf extract (PE) and/or natamycin (NA). Both the addition of PE and NA to the film caused an increase in antioxidant activity. On the other hand, the addition of PE and NA to cassava starch–chitosan films reduced scavenging activity, which may be attributed to the antagonistic effect associated with interactions between these active ingredients [36]. 

### 2.2. Antimicrobial Agents

The incorporation of antimicrobial substances into the biopolymer matrix becomes a valuable strategy for extending the shelf life of various packaged food products. The potential mechanisms of the active ingredient in the antimicrobial action include: (1) membrane rupture with inhibition of ATPase activity; (2) leakage of necessary biomolecules from the cell; (3) disturbance of the proton motive force; (4) inactivation of the enzyme [46].

When designing this type of packaging, it is necessary to establish balance between the kinetics of microbial growth and the controlled release rate of antibacterial agents. There are four categories of antimicrobial packaging systems: (1) The incorporation of volatile antimicrobial substances into a sachet/pad within the packaging; (2) direct incorporation of an antimicrobial agent into the packaging film, for example, by co-extrusion of synthetic films with antimicrobials. However, heat treatment causes high loss of bioactive compounds, and thus other methods that do not use heat treatment, such as casting, electrospinning or solvent mixing, may be an alternative; (3) Packaging coating with a matrix, such as polysaccharides, that acts as a carrier for the antimicrobial agents, so that the active substances can be released onto the food surface by evaporation into free space (volatile substances) or migration to food (non-volatile substances) through diffusion; (4) The use of polymers that have inherent antimicrobial effects, e.g., chitosan [47,48]. In this section, the effects of active ingredients (plant extract, essential oils, nanofillers and other compounds) on the antimicrobial properties of films based on proteins and polysaccharides are described.

#### 2.2.1. Plant Extract

Various plants may comprise a source of antimicrobial compounds to be used during microbial contamination [49]. Phenolic compounds are more effective against Gram-positive than Gram-negative bacteria [50]. Hannani et al. (2019) obtained fish gelatin films with pomegranate peel powder, which showed greater antimicrobial activity against *S. aureus* (7 mm of inhibition zone) and *L. monocytogenes* (5.13 mm of inhibition zone) than *E. coli* (4.13 mm of inhibition zone) [14]. Similar results were observed in gelatin–polyethylene bilayer films. The addition of pomegranate peel increased the antimicrobial activity against *B. cereus* (from 0 to 12.50 mm of inhibition zone), *L. monocytogenes* (from 0 to 16.50 mm), *S. thyphi* (from 0 to 14 mm) and *E. coli* (from 0 to 8 mm). In this work, other active ingredients were also added to this type of film: papaya and jackfruit. Only in the case of *S. thyphi* did this type of film show activity (papaya from 0 to 14 mm inhibition zone, and jackfruit from 0 to 14.50 mm). Pomegranate peel has polyphenols, in particular tannins, which have the ability to precipitate protein, and this causes leakage of the cell membrane and, consequently, cell lysis [51,52,53]. In many works, the positive effects of plant extract addition on the antimicrobial properties of the investigated films have been demonstrated [54]. The presence of flavonoids (mainly 7-O-β-glucoside luteolin and 7-O-β-glucoside) in *Sonneratia caseolaris L.* caused an increase in the antimicrobial effects of chitosan films against *P. aerugino* [55]. Nouri et al. (2018) noted that the addition of *Rosmarinus officinalis L*. extract to the biopolymer matrix increases the antimicrobial activity of the κ-carrageenan/nanoclay film. Compared to the control film, this type exhibits > 99% inhibition against *B. cereus*, *E. coli*, *P. aeruginosa* and *S. aureus* [21]. 

#### 2.2.2. Essential Oils

Essential oils are a source of bioactive compounds, such as terpenoids and phenolic, that are recognised as antimicrobial agents. Essential oils attack microbial cells through a variety of mechanisms: by attacking the phospholipid bilayer cell membrane, disrupting enzymes, forming fatty acid hydroperoxidase caused by oxygenation of unsaturated fatty acids, and violating the genetic material of bacteria [56,57,58].The inclusion of essential oils in the structure of the film may be associated with a change in its functional properties, because this type of active ingredient can interact with both the polymer and the plasticiser, which reduces diffusion into the product. In addition, essential oils are quite volatile, and many processes, e.g., high temperature processing, can degrade them. A solution to this problem could be microencapsulation or film-forming processes that allow isolation, transport and controlled release. Benavides et al. (2012) noted that the inhibitory effect of oregano essential oil incorporated in alginate film was lower than that of pure essential oil. The reason for this may be the partial loss of volatile compounds during film preparation. In addition, interactions between the hydroxyl groups of phenolic compounds and polymer chains may be the reason for the slower diffusion of essential oil phenolic compounds [59]. The release of antimicrobial agents from the film depends on the electrostatic interactions between the antimicrobial agent and the biopolymer chains, structural changes caused by the presence of the microbial agent, osmosis, and environmental conditions [56,58]. dos Santos Paglione et al. (2019) compared the effects of adding free oregano essential oil and oregano oil in microcapsules on the functional properties of soy protein concentrate films. The results indicated that the soy protein concentrate films with microencapsulated essential oil showed better microbiological activity than the films with free essential oil. However, it should be noted that oregano essential oil, in the form of microcapsules among the film, exhibited lower antioxidant activity than the free oil in the soy protein concentrate film. The authors attribute this to the presence of a solvent, which is used to extract antioxidant compounds and modify the release profile of oils from the film to the release medium [60]. Rosemary essential oil, extracted from two Tunisian varieties, namely Zaghouan (ZG; North Tunisia; *R. officinalis* var. *Typicus*) and Chaab Tweel (CT; South Tunisia; *R. officinalis* var. *Troglodiytarum*), was added to the gelatin–chitosan–pectin film. Improvement in antimicrobial activity was observed against *B. subtilis*, *S. aureus*, *E. aerogenes*, *E. faecalis* and *E. coli.* These results indicated that the films with ZG essential oil had higher activity compared to films with CT [61].

#### 2.2.3. Nanofillers

The nanostructures added to the biopolymer matrix had an influence on the antimicrobial activity of the films. Amjadi et al. (2019) prepared different types of gelatin films with zinc oxide nanoparticles (ZnONPs) and/or chitosan nanofiber. The gelatin films and those with chitosan nanofiber did exhibit any heavy antimicrobial activity. The addition of ZnONPs caused improvement in the antimicrobial effects of gelatin films against *E. coli*, *S. aureus* and *P. aeruginosa*. Higher values of the inhibition zone could be observed in gelatin films with ZnONPs + chitosan nanofiber, which proves the synergistic effect of ZnONPs and chitosan nanofiber on the antimicrobial properties of the gelatin matrix [62]. The type of nanofiber in the nanohybrid also affects the antimicrobial activity of the film. Almasi et al. (2018) noted that the addition of bacterial cellulose nanofiber decreased the antimicrobial activity of CuONPs, while chitosan nanofibres showed no synergistic effect on the antibacterial activity of CuONPs [63]. Peighambardoust et al. (2019) created active starch-based films, incorporating a combination of Ag, ZnO and CuO nanoparticles. The starch films with single nanoparticles (NPs) showed antimicrobial activity against *S. aureus* and *E. coli*, but starch films with a combination of NPs had better antimicrobial properties. Potential antimicrobial mechanisms are based on the synergistic action of NPs, and involve the destruction of the cell wall and bacterial DNA damage. Damage to DNA can lead to the disruption of microorganism replication and damage to the ribosome, thereby blocking the production of energy cycle enzymes [64]. The synergistic effect of NPs may consist in the use of various microbial mechanisms by specific NPs. When one type of NPs damages the cell membrane, the other type of NPs can affect the DNA of bacteria [65].

There are two techniques for producing nanocomposite films. The first method (in situ) involves the use of a biopolymer matrix as a reaction medium to form nanostructures, acting on them as a stabilising agent. The second method (ex situ) is used as a dispersion and stabilising medium for separately pre-synthesized nanostructures [66]. Biopolymers can also constitute a matrix for obtaining stable nanostructures, while eliminating the use of environmentally harmful solvents and reducing agents. Such a matrix may be negatively charged furcellaran, which is obtained from *Furcellaria lumbricalis* red algae. This sulfated polysaccharide was used to obtain silver nanoparticles (AgNPs) with antibacterial activity against *E. coli*, *E. facealis*, *P. aeruginosa*, *S. aureus* and *C. albicans.* Moreover, nanocomposite films prepared using environmentally-friendly methods showed improved barrier properties against UV radiation and water vapour, as well as very good mechanical properties [67]. The use of nanofillers in films not only affects the active properties of packaging materials, but also has an influence on the controlled release of active substances from biopolymer films. The addition of halloysite to corn starch films, with peptides nisin and pediocin, caused the inhibition zone to be smaller than in the case of active films without halloysite. The presence of this type of nanofiller affects the controlled diffusion or increase of antimicrobial agents by biopolymer matrices [68,69]. 

In Table 2, information is presented regarding recent studies on active biopolymer films with antimicrobial activity.

#### 2.2.4. Other Antimicrobial Compounds

The cottonseed protein hydrolysate in alginate films has an inhibitory effect against *S. aureus*, but it does not exhibit any influence on *E. coli* [30]. The difficulty in accessing bacteria may result from their different morphology. Gram-negative bacteria have a cellular envelope consisting of lipopolysaccharide molecules that act as a barrier [30]. Oleoresin spices have resins in their compositions, and are used in food formulations [77]. Likewise, this type of natural active compound could be used as an antimicrobial agent in food packaging materials [78]. Oleoresin cloves have greater antimicrobial activity against *E. coli* and *S. aureus* than nutmeg and black pepper oleoresins when used in gelatin films [78]. Another interesting active additive to the biopolymer matrix is castor oil. Its addition to alginate films affects their antimicrobial properties. The authors reported that castor oil increases the antimicrobial properties of the film against Gram-positive bacteria (*S. aureus*—from 0 to 16.97 mm of inhibition zone; *B. subtilis*—from 0 to 17.30 mm), which is a result of the increased presence of hydroxyl groups affecting the hydrophilic nature of the films. This property facilitates the dissolving processes in the bacterial membrane, and then aids in the repair of damage. However, this type of film was unable to cope with the extra outer membrane of Gram-negative bacteria (*E. coli* and *S. typhi*) [79].

To provide the packaging with increased active properties, a mixture of antimicrobial additives is often used. Sani et al. (2019) developed packaging materials based on chitosan, melissa essential oil and ZnONPs. The presence of citronellal and geraniol compounds in the essential oil affects the antibacterial properties of the packaging material, because the compounds destroy the outer membrane of microorganisms and cause the release of liposaccharides, while increasing the permeability of the cytoplasmic membrane to ATP, which consequently causes cell death [80]. Cumin essential oil (CEO) and titanium dioxide (TiO_2_NPs) were added to the sodium caseinate–agar film in various concentrations and combinations. The activity of the biopolymer films varied depending on the used active additive and the type of bacteria. Films containing 1% TiO_2_NPs demonstrated better activity against *E. coli*, *S. enteritidis*, *L. monocytogenes* and *S. aureus* than films with 2% CEO. In contrast, films with 1% TiO_2_NPs and 2% CEO exhibited a synergistic effect on antimicrobial activity. This type of CEO activity is associated with the presence of phenolic compounds, such as 1,8-cineole, 4-dien-7-al, cumin aldehyde, γ-terpinene and β-pinene, which have hydrophobic and lipophilic functional groups. In contrast, the activity of TiO_2_NPs is assigned to the crystal structure, shape, size, and large area to volume ratio [81]. The presence of carvacrol and thymol in *Ziziphora clinopodioide* essential oil, and lavanols, procyanides and phenolic acid in grape seed extract, was the reason for the increase in the antimicrobial properties of chitosan and gelatin films against *L. monocytogenes*, *S. aureus* and *B. cereus* [82]. 

Furthermore, the incorporation of active ingredients improves the antifungal properties of biopolymer films. The addition of cottonseed protein hydrolysate to alginate films caused antifungal activity against *C. gloeosporioides* (14.4–27.02 mm of inhibition zone) and *R. oligosporus* (11.03–25.91 mm) [30]. The antibacterial mechanism of peptides is based on triggering changes in the biological membranes through the formation of ion channels. These changes cause deregulation of the replication, transcription and DNA sequence translation processes, with a disturbance to intracellular balance [30,83]. Dairi et al. (2019) obtained cellulose acetate/AgNPs–montmorillonite films with or without thymol. Antifungal tests against *A. niger* showed the best effects of ternary films. The presence of thymol caused a reduction in fungal colony density. The authors stated that nanoclay can control the release of thymol by retarding its vaporisation process [84]. Moreover, antifungal turmeric oil was added to the chitosan film, which showed an inhibitory effect against *Aspergillus flavus*. Essential oils can cause morphological changes in fungal hyphae. It is worth noting that the chitosan matrix slowed down the process of oil release due to an interaction between the oil and the biopolymer [85]. Adding chitin nanoparticles to gelatin films initiated an antifungal effect against *Aspergillus niger*. Sahraeet et al. (2017) assigned two potential mechanisms of antimicrobial activity to chitin nanoparticles. First, the nanoparticles penetrate the cell membrane, bind to DNA and block the synthesis of RNA and proteins. The second mechanism is based on the interaction of positively charged amino groups of chitin, chitosan and their derivatives with negatively charged groups on the microbial cell membrane, which disturbs its functionality [86].

## 3. Studied Systems in the Food Industry

The food industry develops various types of packaging, designed to reduce losses associated with spoilage of food products. In recent years, active packaging has become an alternative to synthetic packaging materials. There are various reports in the literature on the subject of the influence of active biopolymer coatings on food products. This type of packaging material demonstrated a protective function during the storage of fruit [87,88,89,90,91], meat [92,93,94,95], vegetables [96,97], cheese [98,99], fish and seafood [100,101,102,103].

### 3.1. Plant Extracts

One of the methods used for measuring oxidation level and oil degradation is the peroxide formation index, or peroxide value (PV), which is formed as a result of saturation of unsaturated fatty acids. Due to the presence of saturated hydrocarbons, aldehydes and ketones, physical and chemical changes in the food product occur, which result in an unpleasant smell and taste [104]. Rambabu et al. (2019) obtained chitosan films with mango leaf extract (MLE) in different concentrations (1%, 3% and 5%), and used them for the preservation of cashew nuts. Chitosan films with 3% and 5% MLE were characterised by a significant increase (56% higher than chitosan films without extract) in resistance to oxidation during the 28-day storage period of cashew nuts. A 62% increase of resistance was recorded for the minimal oxidation effects of films with 5% MLE [104]. Malherbiet al. (2019) added guabiroba pulp to corn starch/gelatin films, to be applied as packaging for extra-virgin olive oil. After 15 days of storage, the peroxide index for the samples wrapped in corn starch/gelatin and guabirola pulp films did not reach the maximum limit allowed by Brazil’s legislation, however, the addition of guabirola pulp did not have an additional effect on the oxidative stability of the extra virgin olive oil [105]. 

### 3.2. Essential Oils

Encapsulation of essential oils overcomes the problems associated with the oxidative stability of bioactive compounds and the provision of regulated or targeted transport. Lemon essential oil was encapsulated in chitosan nanoparticles, and was then placed in gelatin films in this form. The obtained active packaging materials effectively prevented the proliferation of microorganisms, inhibited lipid oxidation and delayed the deterioration of pork for 21 days at 4 °C [106]. Zhang et al. (2020) noticed that tarragon essential oil in the form of nanocapsules placed in chitosan–gelatin films caused a prolonged storage of pork slices in refrigerated conditions, compared to chitosan–gelatin films enriched with tarragon oil alone [107].

The content and type of biopolymers are of great importance when designing active packaging materials. Lian et al. (2020) obtained active packaging materials based on chitosan and two antimicrobial polysaccharides (xanthan and pullulan) or polysaccharides of plant origin (gum tragacanth and arabic gum). Then, they examined the effect of the polysaccharides used on the release effect of thyme essential oil film. The application of these types of film in the packaging of nectarine fruits was also investigated. After 60 h of storage, chitosan–gum arabic film had the best inhibitory effect. The addition of gum arabic to the chitosan–thyme oil film strengthened antifungal activity, and delayed the release of the oil by the electrostatic interaction between polysaccharides and the emulsifying effect of gum arabic on essential oil. Moreover, the release of essential oil can be controlled by regulating interactions between polysaccharides [108].

In Table 3, the impact of using active biopolymer films on the storage quality of various food products is presented.

### 3.3. Nanofillers

One of the possibilities for improving the functional properties of biopolymer films is the addition of nanofillers. However, there is a tremendous fear that nanofillers will affect the quality of packaged food products. There are works that touch upon this aspect. Salarbashi et al. (2018) obtained soluble soybean polysaccharide films with the addition of TiO_2_NPs, and then checked whether TiO_2_NPs were transferred to the food products (in bread, for a 6-month storage period). The authors found that TiO_2_NPs were in concentrations acceptable by health requirements. In addition, a low incidence of TiO_2_NPs was found, as low concentrations of nanoparticles were detected in rat intestine epithelial cells [145]. Nonetheless, further research is needed to exclude the negative effect of TiO_2_NPs on human health. The influence of gelatin–carboxymethyl cellulose (Gel–CMC) films, with the addition of chitin nanofibers (CHNF) and *Trachyspermum ammi* essential oil (AJEO), on the quality of raw beef during 12 days of storage, at a temperature of 4 °C, was assessed. Active packaging materials caused delayed lipid oxidation and decomposition of proteins. The initial population, with a total viable count (TVC) in the control sample (without active film) of 3.1 log CFU/g, increased to 9.4 log CFU/g after 15 days of refrigerated storage. The lowest TVC values were recorded in samples wrapped in active films, which totaled 4.5 log CFU/g (in films with 4 wt % CHNF and 1% vol. AJEO) and 5.1 log CFU/g (in films with 2 wt % CHNF and 1% vol. AJEO). In addition, films with 2 wt % and 4 wt % CHNF, as well as 0.64% and 1% vol. AJEO, effectively inhibited the psychrotrophic bacterial count (PTC) in raw beef. After the storage period, the lowest increase in the total count of *Pseudomonas *spp. was recorded for beef wrapped in active films with 4 wt% CHNF and 1% vol. AJEO (from 1.94 to 3.05 log CFU/g). The presence of CHNF in the biopolymer matrix may act as a good oxygen barrier, which, in turn, can prevent the growth of aerobic bacteria. In addition, the presence of this type of nanofiller, due to its internal antimicrobial activity, reduces yeast and mould growth in packaged beef samples. Active Gel–CMC films reduced the TVB-N of raw beef, and after 15 days of storage, the content remained below the standard appropriate range, according to the TVB-N acceptance limit for beef samples (16.5 mg N/100 g). Gel–CMC films, with the addition of 4 wt% CHNF and 1% vol. AJEO, can extend the storage of beef, leading to minimised colour changes [146]. Wang et al. (2019) obtained anthocyanin nanocomplexes, produced using chitosan hydrochloride as well as carboxymethyl chitosan, and then incorporated them into gelatin films. The nanocomposite obtained in this way significantly delayed the deterioration of olive oil oxidation (21.2 meq O_2_/kg of peroxide value on the 56th day) compared to samples with gelatin films (24.3 meq O_2_/kg of peroxide value on day 56) [147].

### 3.4. Other Compounds

There are many scientific studies that focus on assessing the impact of active packaging enriched with antioxidant and/or antimicrobial agents on the properties of food products during storage. The addition of rosemary acid (RosA) to the gelatin film significantly affected the storage quality of Chinese bacon. After 60 days of storage, the difference in PV between the bacon in the gelatin film and the samples with the addition of 0.08% RosA was ~31%. Therefore, due to the presence of four phenolic-OH groups in RosA, lipid oxidation was delayed in the Chinese bacon [135]. Various bioactive additives were included in the starch films (gallic acid, chitosan and carvacrol), and their effect on ham was then examined during a 28-day storage period at 4 °C. The films with gallic acid inhibited the antimicrobial activity of ham the least. This was due to the presence of three hydroxyl groups in the given type of acid, while films with chitosan and carvacrol had an inhibitory effect on the growth of *L. monocytogenes* throughout the entire storage period. Furthermore, starch films enriched in chitosan or chitosan and carvacrol delayed the growth of the meat microflora of ham by 1 to 2 weeks. Antimicrobial activity of hydroxybenzoic acid decreases along with the increase in the number of hydroxyl groups, as hydroxyl groups increase polarity, and thus reduce membrane diffusion [148]. Zhang et al. (2020) studied the effect of a chitosan–zein film, with α-tocopherol, on the physicochemical properties and enzymatic activity of mushrooms (*Agaricus bisporus*) during storage at 4 °C for 12 days. The results of the study indicated a reduction in weight loss, maintaining firmness of the mushrooms wrapped in active films [149].

Biopolymer films without active additives can also be used as active packaging during the storage of food products. Martinez et al. (2018) used resveratrol as an active additive while storing processed smoked sea bass (*Dicentrarchus labrax*) products. The experiment was designed in several stages: (1) liquid resveratrol was superficially added to smoked fish; (2) resveratrol was suspended in liquid smoke, which was used for immersion of fillets; and (3) some fillets were coated with alginate or chitosan films. The fillets preserved by the first and second methods had reduced TBARS value. Active biopolymer films helped delay the oxidation process, however, only chitosan films were able to almost completely inhibit the growth of mesophilic, psychophilic and anaerobic bacteria. In a recent sensory evaluation, it was indicated that alginate films were conducive to limiting the deterioration of the fish’s condition [150].

Combining active compounds in biopolymer films, and then performing their assessment as packaging materials for food products, was also tested. Grape seed extract (GSE) (1% and 2%) and *Ziziphora clinopodioide* (ZEO) essential oil (1% and 2%) were incorporated in the chitosan–gelatin film. Then, it was checked how active packaging affects the storage of minced trout fillet. For 11 days of refrigerated storage, the active film caused a delay in fish deterioration. The lowest PV and total volatile basic nitrogen (TVB-N) values were observed in fish wrapped with 2% ZEO and 2% GSE films, which exhibited the best organoleptic values [151]. Pomegranate peel extract and *Thymus kotschyanus* essential oil were added to chitosan–starch films, and it was then further examined how this type of active film affects the storage quality of beef during a period of 21 days at 4 °C. The films containing 1% extract and 2% essential oil were characterised by the best results of sensory evaluation, including smell, colour and general acceptability. In addition, after 21 days of storage, this type of film showed the best inhibitory effect against *L. monocytogenes* (from 5.04 to 8.11 log CFU/g), bacterial count (count values of lactic acid bacteria from 3.2 to 3.98 log CFU/g) and lipid oxidation (TBARS values from 0.9 to 1.02 mg malondialdehyde/kg) [152]. *Ziziphora clinopodioide* essential oil alone, or in combination with *Ficus carica* extract (FCH), was added to nanomontmorillonite–chitosan (MMT–CS) and nanomontmorillonite–carboxymethyl cellulose (MMT–CMC) films to improve the quality of camel minced meat during refrigerated storage. After storage, the active films (CMC–MMT + 2% ZEO + 1% FCH and CS–MMT + 2% ZEO +1% FCH) reduced the final meat microbial population by approximately 1–4 log CFU/g, compared to the control film. For meat wrapped in active films, there tended to be a retardation in the growth of TVB-N (from 8.1 to 20 mg of TVB-N/100 g), pH (from 5.9 to 6.1), PV values (from 0 to 0.55–0.63 meq peroxide oxygen/1000 g lipid) and TBARS values (from 0.32 to 1.1–2.91 mg MDA/kg), as well as protein carbonyl content (from 0.72 to 1.08–1.16 nmol/mg protein) [153]. Active films based on cassava starch, pumpkin extract (PRE) and oregano essential oil (OEO) were obtained. Then, the films were used to protect ground beef from oxidation. After 9 days of storage, films containing 2% OEO and 3% PRE demonstrated the lowest 2-thiobarbituric acid reactive substance (TBARS) values compared to the control films ( for those with active ingredients—from 1.9 to 6.1 mg MDA/kg sample; for those without active ingredients—from 1.9 to 8.9 mg MDA/kg sample; MDA = malondialdehyde) [154]. 

## 4. Concluding Remarks and Future Developments

Traditional packaging systems for food products come from non-renewable fossil resources and their disposal poses a problem. Biopolymers are a good alternative because they are biodegradable, available, non-toxic, and also reduce the use of fossil fuels. The combination of biopolymers with antioxidant and/or antimicrobial ingredients creates active and ecological packaging systems.

From the authors’ assessment, future research trends in the field of active packaging systems may include the following areas:

1. Assessing the impact of active packaging on the sensory quality of tested food products. As discussed earlier in this paper, various substances are added to the film that, in addition to antimicrobial and/or antioxidant activity, affects the sensory quality of the products. Research in this area is essential for developing packaging with an optimal active ingredient content that does not adversely affect the sensory properties of the products.
2. Enriching the biopolymer film with nanofillers is combined with the need to develop a mechanism to migrate these types of active additives from the film to the food product. It is also necessary to evaluate the optimal level of nanofillers that can be safely used as an additive to biopolymer films, without adversely affecting human health.
3. More research is required that would focus on understanding the potential mechanisms for combining different active ingredients with various biopolymer matrices, which could help optimise the composition of active films.
4. Another key aspect in further research is the stability of antioxidant and antimicrobial components during storage of active films, and their release during the storage of packaged food.
5. To effectively implement active packaging systems, multi-level cooperation between scientists from various fields (e.g., microbiology, food technology, etc.) and the packaging industry is indispensable.
6. The future of the food and packaging industry involves the development of ‘smart’ packaging systems, that have both active (extending the shelf life of food) and intelligent (conveying information about the quality of the food product) properties.
7. Despite the fact that there is extensive literature concerning the impact of active biopolymer packaging on the quality of food in laboratory conditions, it is necessary to test this type of packaging material in large-scale research, and to develop potential commercial applications.

## Figures and Tables

**Table 1:** Recent studies on biopolymer films with antioxidant activity.

Active Agents	Type of Biopolymer Matrix	Concentration of Active Agents	Antioxidant Activity	Ref.
**Protein Films**				
pomegranate peel powder	fish gelatin	1% to 5%	from~59.74% to ~71.82% (DPPH^·^ method) from~48.40% to ~80.02% (ABTS^+^ method)	[14]
*Centella asiatica* (L.) urban extract	bovine gelatin type B	5% to 25%	from ~31.21% to ~47.51% (DPPH^·^ method)	[27]
*Aloe vera* gel	fish gelatin	1% to 9%	up to ~145% (DPPH^·^ method) up to ~100% (ABTS^+^ method)	[16]
*Morinda citrifolia* oil	fish gelatin	1% to 3%	up to ~3975% (DPPH^·^ method) up to ~7400% (TPC method)	[28]
melanin nanoparticles	gelatin	0.25% to 1%	up to ~996% (ABTS^+^ method) up to ~891% (DPPH^·^ method)	[29]
- montmorillonite (MMT) - clove essential oil (CEO)	soy protein isolate (SPI)	MMT—0.25 g and 0.5 g CEO—0.5 mL	the addition of MMT caused an increase in antioxidant activity of SPI films (up to ~87%—ABTS^+^ method; ~400%—FRAP method; ~193%—TPC method) the addition of CEO caused an increase in antioxidant activity of SPI-MMT films (up to ~2320%—ABTS^+^ method; ~8138%—FRAP method; ~3294%—TPC method)	[26]
**Polysaccharide Films**				
cotton seed protein hydrolysates	alginate	0.15% to 0.60%	up to ~96.9% (DPPH^·^ method) up to ~761.4% (ABTS^+^ method) up to ~950% (FRAP method)	[30]
fungal extract from *Tricholoma terreum*	chitosan	100 mg	up to ~121% (DPPH^·^ method)	[31]
- Montmorillonite (MMT) - Pomegranate rind powder extract (PRP)	chitosan	MMT 1 to 5% PRP 1–2%	films with 3% MMT and 2% PRP had the highest amount of total phenolic (15.2 mg GAE/g DW) and were the most active radical scavengers (~71%—DPPH^·^ method)	[32]
curcumin zinc oxide nanoparticles (ZnONPs)	carboxymethyl cellulose	curcumin 0.5% and 1% ZnONPs 1%	up to ~95% and ~400% in films with ZnONP addition (DPPH^·^ and ABTS^+^ method, respectively) up to ~2016% and ~6067% in films with curcumin addition (DPPH^·^ and ABTS^+^ method, respectively) no synergistic effect was noted in antioxidant properties after adding ZnONPs and curcumin to the film	[33]
melanin nanoparticles	cellulose nanofibres	0.25% to 2%	up to ~1950% (ABTS^+^ method)	[34]
**Polysaccharide-Protein Films**				
peanut shell and skin extracts	starch/chitosan	10, 30 and 60 mL	In Proceedings of the	[11]
pu-erh and green tea extracts	furcellaran/bovine gelatin	5% to 20%	For films with pu-erh extract: from 39.58% to 48.94% (DPPH^·^ method) from 6.84% to 14.98% (ABTS^+^ method) up to ~820% (TPC method) For films with green tea extract: from 42.53% to 48.94% (DPPH^·^ method) from 8.81% to 15.76% (ABTS^+^ method) up to ~310% (TPC method)	[35]
cinnamon essential oil	chitosan/gum arabic	8%	Depending on the ratio of chitosan (CS) to gum arabic (GA), (CS:GA—1:0; 1:0.25; 1:0.5; 1:1; 1:2; 1:4): from 17.32% (at 1:0 ratio) to 44.53% (at 1:2 ratio) (DPPH^·^ method) from 12.63% (at 1:0 ratio) to 40.27% (at 1:2 ratio) (H_2_O_2_ method)	[24]
pitanga (*Eugenia uniflora* L.) leaf extract (PE) and/or natamycin (NA)	cassava starch/chitosan	2.25 g PE/100 g film solution 1 g NA/100 g film solution 2.25 g PE + 1 g NA/100 g film solution	For films with PE: up to ~680% (ABTS^+^ method) up to ~2450% (DPPH^·^ method) up to ~2134% (FRAP method) For films with NA: up to ~87% (ABTS^+^ method) up to ~663% (DPPH^·^ method) up to ~100% (FRAP method) Films with PE + NA: up to ~385% (ABTS^+^ method) up to ~2017% (DPPH^·^ method) up to ~1316% (FRAP method)	[36]
quercetin-starch	chitosan/gelatin	0.16 g quercetin	up to ~299% (ABTS^+^ method) up to ~161% (DPPH^·^ method)	[37]

Abbreviations: DPPH—(2,2-diphenyl-1-picryl-hydrazyl) free radical method; ABTS+—ABTS (2,2’-azino-bis(3-ethylbenzothiazoline-6-sulfonic acid) radical scavenging assay; FRAP—Ferric reducing antioxidant power; TPC—Total phenolic content.

**Table 2:** A summary of biopolymer films with antimicrobial activity.

Active Agents	Type of Biopolymer Matrix	Concentration of Active Agents	Antimicrobial Activity	Ref.
chitosan/gallic acid NPs	konjac glucomannan	5–15%	↑ in antimicrobial activity against *S. aureus* (up to ~20 mm of inhibition zone) and *E. coli* (up to ~13 mm) at 15% NPs	[70]
cinnamon essential oil	fish gelatin	0.5–6%	↑ in antimicrobial and antifungal activity against *E. coli* (up to ~39 mm); *S. aureus* (up to ~40 mm); *A. niger* (up to ~55 mm); *R. oryzae* (up to ~70 mm) and *P. variotii* (up to ~52 mm)	[71]
summer savory essential oil	carboxymethyl cellulose/agar	0.5–1.5%	↑ in inhibition zone: - *B. cereus* (from 0 to 35.71 mm) - *S. aureus* (from 0 to 47.48 mm) - *L. monocytogenes* (from 0 to 30.67) - *E. coli* (from 0 to 25.01 mm)	[72]
frankincense essential oil	carboxymethyl cellulose/chitosan biguanidine hydrochloride	1–5%	↑ in inhibition zone: - *S. pneumonia* (from 19.68 to 22.56 mm) - *B. subtilis* (from 18.23 to 27.48 mm) - *E. coli* (from 13.5 to 18.48 mm)	[73]
apple peel polyphenols	chitosan	0.25–1%	↑ in inhibition zone: - *E. coli* (from 7.51 to 16.12 mm) - *B. cereus* (from 9.38 to 19.46 mm) - *S. aureus* (from 8.73 to 18.11 mm) - *S. typhimurium* (from 5.82 to 14.47 mm)	[74]
- pomegranate flesh extract (PFE) - pomegranate peel extract (PPE)	κ-carrageenan	1–4%	For κ-carrageenan with PFE: - ↑ in inhibition zone for *E. coli* (5.26–6.37 mm), *Salmonella* (5.22–6.25 mm); *S. aureus* (5.14–5.58 mm); *L. monocytogenes* (5.12–5.65 mm) For κ-carrageenan with PPE: - ↑ in inhibition zone for *E. coli* (5.45–6.98 mm), *Salmonella* (5.57–7.04 mm); *S. aureus* (5.42–6.26 mm); *L. monocytogenes* (5.36–6.42 mm)	[75]
cellulose nanowhiskers	chitosan/xylan	0–16%	- during incubation, the optical density values were suppressed against *E. coli* and *S. aureus*	[76]
- AgNPs - CuNPs - ZnONPs	starch	0.67–3%	- films with Ag, ZnO and CuO NPs significantly reduced the CFU count of *S. aureus* and *E. coli* (within the range of 72–97%) - films with AgNPs (at a concentration of 3%) showed the highest antimicrobial activity against both *E. coli* (by 97.27%) and *S. aureus* (by 95.41%) compared to films with a single type of NPs - starch films with a combination of 3 types of NPs (with a total weight of 2 wt %) most effectively inhibited bacterial growth (reduction within the range of 91–94%) - a 4-fold increase in the antimicrobial activity of Ag-ZnO–CuNPs films against *S. aureus* and a 2-fold increase against *E. coli* compared to using films with single NPs	[65]
- chitosan nanofiber (CHNF) - zinc oxide nanoparticles (ZnONPs)	gelatin	CHNF—10% ZnONPs—5%	↑ in inhibition zone: - *E. coli* (15.06 mm—ZnONPs; 25.06 mm—ZnONPs + CHNF) - *S. aureus* (30.62 mm—ZnONPs; 33.13 mm—ZnONPs + CHNF) - *P. aeruginosa* (10.7 mm—ZnONPs; 12.95 mm—ZnONPs + CHNF)	[62]

**Table 3:** Literature review regarding the use of active packaging materials on food products.

Active Compound	Type of Biopolymer Matrix	Functional Properties of Films	Influence on Food Product	Ref.
**Plant Extracts**				
grape seed extract	chitosan	- ↑ in WVP up to ~11%; in EM up to ~63%; in EAB up to ~32% - ↓ in TS up to ~58% - ↑ in antioxidant activity (12.53–20.45%-DPPH^·^ method) - ↑ in antimicrobial activity against *E. coli*, *L. monocytogenes*, *S. aureus*, *P. aureginosa*	Chicken breast fillets: - after 15 days of storage, samples in chitosan films had the lowest pH, while samples in the films with the extract showed the highest pH (6.66) - active films effectively limited the growth of mesophilic aerobic (to ~5.9 log CFU/g) and coliform bacteria (to ~2.3 log CFU/g) - reduction in oxidation value (to ~3.1 mg MDA/kg—TBARS value)	[109]
chinese chive (*Allium tuberosum*) root extract	chitosan	- ↓ in WS up to ~41%; in WC up to ~35%; in WVP up to ~50%; in TS by to ~43%; in EAB up to ~45% - ↑ in antioxidant activity up to ~488% (DPPH^·^ method) and up to ~ 346% (ABTS^+^ method) - ↑ in antimicrobial activity against *E. coli* (from 4.43 to 16.21 mm of inhibition zone); *B. cereus* (from 6.21–18.79 mm); *S. aureus* (7.13–18.12 mm); *S. typhimurium* (4.11–14.91 mm) - active films showed the highest biodegradability (47.36%)	Soybean oil: - lowest oil resistance ability values for active films (from 0.32% to 0.09%) - during 28 days of storage, a reduction in oxidation of samples with active films was noted (PV—reduction up to ~55%)	[110]
pomegranate peel extract	zein	- ↑ in TS up to ~35%, in EAB up to ~28%, in WS up to ~196% - ↓ in WVTR up to ~52% - ↑ in antimicrobial activity against *E. coli* (17.66–22.33 mm), *P. vulgaris* (15.33–27.67 mm), *P. perfringens* (21.67–32.00 mm), *M. luteus* (14.33–24.67 mm), *E. faecalis* (15.00–26.00 mm), *S. aureus* (15.33–26.00 mm), *S. typhii* (14.67–25.00 mm)	Fresh Himalayan cheese (Kalari/kradi): - after 30 days of storage, cheese with active films demonstrated low oxidation of products (carbonyl content—by ~48%; MDA content—by ~70%) and inhibited spoilage of microorganisms (TBC—from ~3.5 to 0.5 log CFU/g; yeast and mould count—from ~2.5 to 0 log CFU/g; LAB—from ~2.5 to 5.5 log CFU/g)	[111]
durian leaf extract	gelatin	- ↑ in antioxidant activity by 17.6 times (DPPH^·^ method) - retarded oil oxidation (three times more efficiently than a gelatin film)	Durian fruit pulp: - higher loss of fruit weight in the active films than commercial ones - decrease in fruit hardness during storage in active films - no significant changes in resilience value	[112]
*Sonneratia caseolaris* (L.) (SCELE)	chitosan	- ↑ in TS up to ~2%; in WS by up to ~116% - ↓ in EAB up to ~72%, in WVTR up to ~15%	Vietnamese banana fruit: - after 4 days of storage at room temperature, unwrapped bananas had visible black spots, while the fruits with chitosan and chitosan–SCELE films were still yellow	[55]
guabiroba pulp	corn starch/gelatin	- ↑ in WVP up to ~234% - ↓ in TS up to ~82%	Extra-virgin olive oil: - no changes in acidity or peroxide indices (after 15 days of storage, the samples were within the norm, where the maximum acidity index allowed is 0.8% of oleic acid and the maximum peroxide index amount permitted for extra virgin olive oil is 20 meq kg^−1^—Brazil’s legislation)	[105]
coconut husk extract (EECH) Cloisite Na^+^	tilapia and squid skin gelatins (SGF)	-	Mackerel meat powder: - after 30 days of storage, lower PV (~63 mg/kg sample), TBARS (~17 mg/kg sample), TVB content (105 mg N/100 g sample) and pH values were observed for the SGF–Na-EECH sample than the PE sample or the control - the sample with SGF-Na-EECH had the lowest content of volatile lipid oxidation products	[113]
maqui berry extract	cowpea starch	- ↑ in antioxidant activity (from 0% to 88.46%—ABTS^+^ method; from 0% to 42.39%—DPPH method) depending on concentration - ↓ in TS up to ~52% - ↑ in EAB up to ~102%; in WC up to ~29%; in WS up to ~ 29%; in WVP up to ~22%	Salomon: - after 6 days of storage, the PV value for unpackaged samples increased up to ~341%, while samples with active films increase up to ~104%. - the TBARS value for unpackaged samples increased by 35%, while for those with active films, the increase was by 15%	[114]
*Prunus maackii* extract (EPm)	κ-carrageenan/ hydroxypropyl methylcellulose (κC/Hm)	- ↓ in WVP (up to ~20%); in TS (up to ~30%) - ↑ in EAB (up to ~23%); in heat-sealing strength (up to ~124%); in tearing force (up to ~48%); in antioxidant activity (23.75%- DPPH^·^ method); in OP (up to ~95%)	Lard: - after 15 days, the POV and acid values of the lard packaged in κC/Hm/EPm films (4.95–9.37 mmol/kg, 1.01–1.50 mg/g, respectively) were significantly lower than those in the PE film (85.76 mmol/kg and 1.85 mg/g, respectively) - κC/Hm/Epm films with the highest concentration of the extract (8%) were visually sensitive to volatile nitrogen, therefore, they can be used as an intelligent indicator to monitor freshness of lard	[115]
**Essential Oils**				
*Eucalyptus Globulus* essential oil	chitosan	- ↓ in WC (up to ~44%); in WS (up to ~39%); TS (up to ~28%) - ↑ in WVP (up to ~159%); EAB (up to ~66%) - antimicrobial effect (in liquid medium, reduction by 4.22 log *(S. aureus*), 3.98 log (*E. coli*), 4.55 log (*B. cereus*) and 4.71 log (*S. entertidis*)	Sliced sausage: - samples coated by films with 0.5%, 1% and 1.5% oil showed growth inhibition against *L. monocytogenes* by 0.26, 0.7 and 1.01 log (CFU)/mL, respectively	[116]
thyme essential oil	chitosan	-	Ready-to-eat meat: - over 4 weeks, active films showed reduced yeast populations, whereas aerobic mesophilic bacteria, LAB count and enterobacteria were not affected - reduction in water condensation inside the package	[117]
oregano essential oil	gelatin–chitosan	- ↓ in TS (up to ~33%); WS (up to ~8%); WVP (up to ~18%) - ↑ in EAB (up to ~132%)	Grass carp muscle: - after 12 days of storage at 4 °C, TVB-N values of samples with active films (from 7.84 to 40 mg N/100 g) were much lower than parafilm-packaging and control samples (from 7.84 to 55 mg N/100 g) - TPC values for samples with active films (from4.23 to 6.9 log CFU/g) were much lower than parafilm-packaging and control samples (from 4.23 to 7.4 log CFU/g) - pH values of samples in active films decreased from 7.11 to 6.98, while in parafilm-packaging and control samples, there was an increase to 7.32–7.34	[118]
lemongrass essential oil	gelatin	-	Sea bass slices: - after 12 days of storage, samples wrapped in active films showed delayed growth of TVC (from 4.5 to 5.6 log CFU/g), LAB (from 0 to 5.9 log CFU/g), psychrophilic bacteria (from 2.2 to 4.0 log CFU/g) and spoilage microorganisms including H_2_S-producing bacteria (from 2 to 2.2 log/CFU/g) and *Enterobacteriaceae* (from 3.6 to 4.5 log CFU/g) - reduced changes were observed in colour, K value, TVB value (from 12.47 to 15.84 mg N/100 g) and TBARS value (from 2 to 2.1 mg MDA/kg) compared to control films	[119]
*Syzygium aromaticum* essential oil	corn starch	-	Sausages: - after 15 days of storage, reduction in lipid oxidation (TBA value—from 0.05 to 0.23 mg MAL/kg)	[120]
- clove essential oil - cinnamon essential oil	corn starch	- ↑ in antimicrobial activity against *L. lactis* (inhibition zone 12.6–37.6 mm), *L. monocytogenes* (11.3–36.2 mm), *L. mesenteroides* (14.5–35.1 mm), *P. fluorescens* (9.1–29.2 mm), *S. putrifaciens* (10.8–31.7 mm), *S. typhimurium* (9.7–25.8 mm), *E. coli* (12.8–27.6 mm)	Raw beef fillets: - during 15 days of storage, a decrease in microbiological contamination was noted (reduction TVC—by 0.6–0.9 log CFU g^−1^, LAB—by 0.55–0.65 log CFU g^−1^, *Pseudomomas *spp. counts—By 0.51–0.67 log CFU g^−1^, *Enterobacteriaceae*—By 0.4–05 log CFU g^−1^) - reduction in lipid oxidation (TBARS value from 0.39–0.42 to 1.32–1.49 mg MDA/kg)	[121]
**Nanofillers**				
AgNPs	chitosan/gelatin	- ↓ in TS up to ~27% - ↑ in EAB up to ~51%	Red grapes: - extension of storage period by 14 days	[122]
ZnONPs	chitosan/carboxymethyl cellulose	- ↑ in TS up to ~85% - ↑ in antimicrobial activity against *S. aureus* (from 5 to 11 mm); *B. subtilus* (from 2 to 10 mm); *B. cereus* (from 4 to 10 mm); *P. aeruginosa* (from 3 to 11 mm); *E. coli* (from 3 to 9 mm); *L. monocytogenes* (from 2 to 8 mm); *C. albicans* (from 3 to 15 mm); *A. niger* (from 3 to 12 mm)	Egyptian soft white cheese: - after 30 days of storage, no differences in chemical properties between samples wrapped in active and control films were noted - active films had good influence on total bacterial counts (bacterial counts—from 2.30 to 0 log CFU/g; coliform—from 1 to 0 log CFU/g), mould and yeast (from 1 to 0 log CFU/g) in soft white cheese	[123]
ZnONPs	chitosan/guar gum	- ↑ in TS by to ~168%; in EAB up to 44% - ↓ in OTR up to ~72%; in WVTR up to ~55% - ↑ in antioxidant activity (up to ~351%—measured by Total phenol content method, and by to ~335%— measured via DPPH method) - ↑ in antimicrobial activity against *E. coli* (31.33 mm), *L. momocytogenes*(30.33 mm), *A. terries* (30.67 mm), *A. niger* (24.00 mm), *A. flavus* (31.33 mm), *B. cereus* (21.00 mm), *S. aureus* (24.67 mm), *P. aeruginosa* (27.00 mm), *Y. enterocolitica* (25.33 mm), *S. typhiurium* (29.00 mm)	Ras cheese: - active films protect the cheese surface for about 3–4 months against yeast, mould and other bacteria	[124]
AgNPs+ SeNPs	furcellaran	- ↓ in WS up to ~33%; in WVTR up to ~2% - ↑ in WC up to ~24%; in modulus of elasticity up to ~97%; in EAB up to ~13% - ↑ in antimicrobial activity against *E. coli* (inhibition zone from 0 to ~27 mm); *S. aureus* (from 0 to ~16 mm) and MRSA (from 0 to ~16 mm)	Mini kiwi: - after 8 days of storage, fruits in active films experienced weight loss at the level of ~0.15%, while the fruits in low-density polyethylene (LDPE) films were not suitable for examination - after 6 days of storage, fruits in LDPE films were entirely covered with mycelium, while the fruits in active films had no signs of mycelium	[125]
chitosan thymol NPs	chitosan/quinoa protein	- ↓ in WVP up to ~20%	Blueberries and cherry tomatoes: - inhibition in weight loss (at 9 days, for blueberries—up to ~8% lower than control films, cherry tomatoes—up to 2.1% lower than control films)	[126]
chitosan nanoparticles (CNP)	starch	- ↑ in antimicrobial activity against gram-positive bacteria (*S. aureus* and *B. cereus*) than gram-negative bacteria (*E. coli* and *S. typhi*)	Cherry tomatoes: - after 10 days of storage, the increase in TPC value for tomatoes without films was 114.5 times the baseline amount - the increase in TPC value for tomatoes packed in starch films alone was 20.5-fold - the increase in TPC value for tomatoes in starch films with 15% CNP was 0.86-fold	[127]
nanocellulose	chitosan	- ↑ in antimicrobial activity against *S. aureus*, *E. coli* and *S. entertidis*	Ground meat: - after 6 days of storage, nanocomposite films reduced the population of LAB by ~3.1 log (at 25 °C) and 1.3 log (at 3 °C) cycles compared to meat wrapped in nylon	[128]
- cellulose nanowhisker (CNW) - CuONPs	sodium alginate (SA)	- CNW films (0.5%)—SA (3%)–CuNPs (5 mM) had the best antimicrobial activity against *S. aureus* (27.49 mm), *E. coli* (12.12 mm), *Salmonella* sp. (25.21 mm), *C. albicans* (23.35 mm), *Trichoderma* spp. (5.31 mm) as well as antioxidant activity (~50% in DPPH^·^ method and ~37% in ABTS^+^ method)	Yellow bell pepper (*Capsicum annuum L. var. grossum* (L.) Sendt): - films with CNW (0.5%)—SA (3%)—CuO NPs (5 mM) prevent microbial contamination (such as total bacteria, total fungi, total *Listeria *spp. and total *Salmonella *spp.) in samples up to 7 days	[129]
**Other Active Ingredients**				
- nisin - catechin	gelatin	- the addition of catechin improved antioxidant activity (DPPH^·^ method) - ↓ in TS, EAB and solubility, nisin has antimicrobial activity against *E. coli*, *B. cereus*, while catechin has no antimicrobial effect	Minced pork: - films with nisin and catechin retard microbial growth—TVC count and psychrophilic aerobic bacteria count (from 6.3 to 8.0 log CFU.g^−1^) after 7 days of storage - no changes regarding weight loss of meat in different types of packaging—retarded lipid oxidation of minced pork	[130]
epigallocatechin gallate	gelatin	- ↑ in TS up to ~36% - ↓ in EAB up to ~97%; in WVP up to ~9%; in OP up to ~57%	Chicken skin oil: - after 30 days of storage, samples packed in active films showed lower PV (from ~0.4 to 2.2 mg cumanehydroperoxide equivalent/100 g oil), TBARS (from 0 to ~1.1 mg MDA/100 g oil) and volatile compounds in comparison to those packaged in LDPE films	[131]
- ferulic acid (FA) - *Cymbopogon citratus* essential oil (EO)	cashew gum/gelatin	- ↑ in WVP up to ~83%; in EAB up to 4517% - ↓ in WS up to ~96%; in TS up to ~97%	Bread: - increased permeability of active packaging compared to polyethylene packaging, making the stored bread more stale - for at least 6 days, due to the smaller amount of water, the bread packed in active films was protected against microorganisms	[132]
- tannic acid - caffeic acid - green tea extract	turmeric/gelatin	- ↑ in TS, WVP and WS - ↓ in EAB in every type of active compounds - no changes in antioxidant activity (DPPH^·^ method)	Ground pork: - all films with phenolic compounds delayed lipid oxidation and had no effect on their sensory attributes (after 12 days of storage, TBARS values from 0.1 to 0.6 mg MDA/kg meat)	[133]
ethyl lauroylarginate (LAE)	oxidised cornstarch/bovine gelatin	- ↓ in WVP up to ~12%	Marinated salmon: - after 45 days of storage, TVC in salmon samples remained below the legal limit (106 CFU/g) - no changes in antilisterial activity in salmon samples - films were not effective in controlling weight loss of salmon samples during the cold storage	[134]
rosemary acid (RosA)	rabbit skin gelatin	- ↑ in TS up to ~22% - ↓ in WVP up to ~8% and in WS up to ~26% - ↑in antioxidant activity (DPPH^·^ and reducing power methods)	Chinese bacon: - after 60 days of storage, PV of samples wrapped in gelatin films with 0.08% RosA was 16.86 meq peroxide/kg (while in control films, this value was 27.03 meq peroxide/kg) - the value of TBARS after 15 days of storage for samples in gelatin films (0.584 mg MDA/kg sample) was the same as for samples in films with 0.08% RosA after 45 days (0.586 mg MDA/kg sample)	[135]
- hydroxytyrosol (HT) - 3,4-dihydroxyphe Nyl glycol (DHPG) - beeswax	pectin/fish gelatin	- the addition of beeswax improved OP	Beef meat: - on the 6th day of storage, improvement in lipid oxidation was noted up to 68% (HT) and 59% DHPG—TBARS method - synergistic effect of HT and beeswax in lipid oxidation reduction by 100% over 7 days - the addition of beeswax improved oxidation stability of the stored meat	[136]
- ZnO nanorods - clove essential oil	bovine skin gelatin	- ↑ in TS up to ~16% - ↑ in EAB up to ~110% - ↑ in OP up to ~32%	Peeled shrimp: - after 20 days of storage, the samples packed in active films reduced bacterial growth (by 2 to 3 log for *L. monocytogenes* and *S. typhimurium*)	[137]
- TiO_2_NPs - rosemary essential oil	cellulose nanofiber/whey protein	-	Lamb meat: - slowed PBC growth rate of samples in active films (5.3 log CFU/g after 15 days of storage) compared to the polyethylene bags (7.1 log CFU/g after 9 days storage) - changes in pH values from 5.70 to 7.25 (in polyethylene bags) and 6.18 (in active films) - active films were highly effective at reducing TVB-N values (to 17.26 mg/100 g); PV values (to 1.46 meq peroxide/1000 g); TBARS values (to 1.23 mg MDA/1000 g); FFAs values (to 10.70%) - according to statistical analysis of sensory characteristics (general acceptability), the shelf life of samples in polyethylene bags was 6 days, while in active films 12–15 days	[138]
- TiO_2_NPs - rosemary essential oil	whey protein isolate/cellulose nanofiber	- ↑ in antimicrobial activity against *L. monocytogenes* (inhibition zone ~19 mm); *E. coli* (~16 mm); *S. aureus* (~19 mm); *P. fluorescens* (~16 mm); *S. enteritidis* (~15.3 mm)	Fresh lamb meat: - after 15 days of storage, active films significantly reduced the bacterial counts of meat samples (TVC—from 4.1 to 6.7 log CFU/g; *Pseudomonas *spp. count—from 2.8 to 5.6 log CFU/g; *Enterobacteriaceae* count—from 1.2 to 6.3 log CFU/g; LAB—from 2.6 to 5.8 log CFU/g; *L. monocytogenes*—3.8 log CFU/g, *E. coli*—6.3 log CFU/g, and *S. aureus*—4.6 log CFU/g	[139]
- montmorillonite (MMT) - clove essential oil (CEO)	soy protein	-	Muscle fillets from bluefin tuna: - active films with MMT and CEO caused a decrease in microbial growth (30–33 mg TVB-N/100 g and microorganism counts—TVB, Total aerobic mesophiles, H_2_S-producer microorganisms, Luminescent colonies, *Pseudomonas *spp., LAB, *Enterobacteriaceae*) and lipid auto-oxidation (evaluated according to the TBA index, during the last 3 days of storage, reaching 0.72 mg MAL/kg muscle) of tuna fillets during the 15-day storage period - the presence of MMT promotes the release of CEO active ingredients by prolonging antimicrobial and antioxidant activity	[140]
- okra mucilage (OM) - ZnONPs	carboxymethyl cellulose	-	Chicken breast meat: - after 12 days of storage, using active films resulted in a reduction in growth rate of TVC values (from 4 to 5.86–6.10 log CFU/g); LAB values (from 2.16 to 2.83 log CFU/g); TBA values (from 0.02 to 0.15–0.26 mg MDA/kg); TVN values (from 12 to 23–35 mg N/100 g) - the odour acceptability level for meat in active films was reached up to the 12th day of storage	[141]
- Zataria multiflora oil (ZEO) - cinnamaldehyde (CIN) in nanoemulsion (NZEO)	corn starch	- ↓ in TS (up to ~74%) - ↑ in EAB (up to ~13%)	Ground beef samples: - after 20 days of storage, samples with NZEO films had better values for PV (3.70 meq/kg of lipid), TBARS (1.03 mg MDA/kg sample), carbonyl content (0.83 nmol/mg protein) and sensory analysis (overall acceptability: 5.85)	[142]
- cellulose nanocrystal - grape Pomace Cabernet Franc (red variety) - grape pomace Viognier (white variety)	tapioca starch	- ↑ in TS up to ~129%; in EAB up to ~104%; in WVP up to ~51% - ↑ in antioxidant activity (within range of 6.67–12.1%—TPC method) - ↑ in antimicrobial activity against *L. monocytogenes* (until 62.7% in growth inhibition) and *S. aureus* (until 97.6% in growth inhibition)	Ready-to-eat chicken meat: - starch/cellulose nanocrystal/Viognier films were the most effective against *L.* *monocytogenes* (1 to 2 log CFU/cm^2^) reduction inoculated on the meat samples during the 10-day storage period at 4 °C	[143]
capsaicin supported by Fe^III^ doped hollow metal-organic frameworks	gelatin/chitosan	- ↑ in TS up to ~12%; in WVP up to ~45%; in WS up to ~36% - ↓ in EAB up to ~32%	White apple cubes: - on the 5th day of storage, apple cubes covered by films with 8% capsaicin content remained almost unchanged (no black colour and only slight water loss)	[144]

Abbreviations: TBARS—2-thiobarbituric acid reactive substance; TVB-N—Total volatile basic nitrogen; TBA—Thiobarbituric acid index; MDA—Malondialdehyde; PBC—Psychrotrophic bacteria count; FFA—Free fatty acid (% Oleic acid) OP—Oxygen permeability; OTR—Gas transmission rate; WVP—Water vapour permeability; WVTR—Water vapour transmission rate; EM—Elastic modulus; TS—Tensile strength; EAB—Elongation at break; PV—Peroxide value; TVB—Total volatile base; PE—Polyethylene films; TPC—Total plate count; TVC—Total viable count; LAB—Lactic acid bacteria; TVN—Total volatile nitrogen.
